# Therapeutic Effects of Astragaloside IV on Myocardial Injuries: Multi-Target Identification and Network Analysis

**DOI:** 10.1371/journal.pone.0044938

**Published:** 2012-09-17

**Authors:** Jing Zhao, Pengyuan Yang, Fan Li, Lin Tao, Hong Ding, Yaocheng Rui, Zhiwei Cao, Weidong Zhang

**Affiliations:** 1 Department of Natural Medicinal Chemistry, College of Pharmacy, Second Military Medical University, Shanghai, China; 2 Shanghai Center for Bioinformation Technology, Shanghai, China; 3 Department of Pharmacology, College of Pharmacy, Second Military Medical University, Shanghai, China; 4 The Teaching Hospital of Chengdu University of TCM, Chengdu, China; 5 School of Life Sciences and Technology, Tongji University, Shanghai, China; 6 Department of Mathematics, Logistical Engineering University, Chongqing, China; University of South Florida, United States of America

## Abstract

Astragaloside IV (AGS-IV) is a main active ingredient of *Astragalus membranaceus* Bunge, a medicinal herb used for cardiovascular diseases (CVD). In this work, we investigated the therapeutic mechanisms of AGS-IV at a network level by computer-assisted target identification with the *in silico* inverse docking program (INVDOCK). Targets included in the analysis covered all signaling pathways thought to be implicated in the therapeutic actions of all CVD drugs approved by US FDA. A total of 39 putative targets were identified. Three of these targets, calcineurin (CN), angiotensin-converting enzyme (ACE), and c-Jun N-terminal kinase (JNK), were experimentally validated at a molecular level. Protective effects of AGS-IV were also compared with the CN inhibitor cyclosporin A (CsA) in cultured cardiomyocytes exposed to adriamycin. Network analysis of protein-protein interactions (PPI) was carried out with reference to the therapeutic profiles of approved CVD drugs. The results suggested that the therapeutic effects of AGS-IV are based upon a combination of blocking calcium influx, vasodilation, anti-thrombosis, anti-oxidation, anti-inflammation and immune regulation.

## Introduction

*Astragalus membranaceus* Bunge, the dried root of a low shrub, has been widely prescribed in traditional Chinese medicine (TCM) for the treatment of cardiovascular disorders. Over 25% of the 90 recipes in the 2010 edition of the Chinese Pharmacopoeia for cardiovascular diseases (CVD) contain *Astragalus membranaceus* Bunge.

Astragaloside IV (AGS-IV) is one of the main active compounds of *Astragalus membranaceus* Bunge [1]. Experimental studies from several laboratories, including ours, have provided abundant evidence demonstrating the explicit cardiovascular-protective effects of AGS-IV [2]–[6]. *In vivo* animal studies have shown that AGS-IV is protective against isoproterenol-induced myocardial injury [3] and isoproterenol-induced cardiac hypertrophy [2], elevates coronary blood flow and reduces the size of myocardial infarcts after coronary occlusion [4]. *In vitro* experiments suggested that AGS-IV could improve post-ischemic heart function and ameliorate reperfusion arrhythmias [4], attenuate hypoxia-induced cardiomyocyte damage [5], and relax smooth muscle in the aorta from both normal rats and stroke-prone spontaneously hypertensive rats [6]. AGS-IV could also enhance the activity of antioxidant enzymes [2], [4], [5], [7]; reduce the levels of phenylephrine and angiotensin II [6], block calcium influx and intracellular calcium release, and stimulate the NO–cGMP pathway [8]. AGS-IV also exhibits significant anti-inflammatory effects *in vivo* [9].

Dysfunctions of multiple genes and/or their products are implicated in the pathogenesis of complex chronic diseases (e.g., CVD) [10]–[13]. Thus, targeting the entire network should be much more effective than targeting a single protein [14], [15].

In this study, we attempted to investigate the therapeutic mechanism of Astragaloside IV against CVD using a network-based methodology that integrates data of drugs, targets and pathways. The *in silico* program INVDOCK [16] was used to search for putative binding sites for AGS-IV in the 3D structures of the proteins in the signaling pathways known to be affected by FDA-approved drugs for CVD. This analysis revealed 39 putative targets of AGS-IV. Experiments at the protein level were carried out to validate three of these targets. Experiments were also carried out in cultured cardiomyocytes to examine protective action of AGS-IV at the cellular effect level. The protein-protein interactions, drug-target and target-pathway association networks of the 39 putative targets were then constructed to probe for relationships between known drugs, targets and pathways and to evaluate how collective actions arising from these relationships contribute to the therapeutic effects of AGS-IV.

## Results

### Identification of key pathways and candidate protein targets associated with CVD therapy

CVD is a series of diseases with complex etiology involving multiple biological processes or pathways [17], [18]. Using existing cardiovascular drugs as a starting point, we applied a network-based approach to probe possible key pathways involved in the therapeutic actions. The DrugBank [19] includes 174 FDA-approved small molecular CVD drugs that act on 188 protein targets. By mapping these targets onto the KEGG pathways [20], it was found that 131 of the 188 targets appear in a total of 120 pathways, corresponding to 133 FDA-approved small molecular CVD drugs. The target-pathway network and drug-pathway network were constructed to reflect the target-pathway and drug-pathway interactions. The distribution of pathway nodes in both target-pathway and drug-pathway networks obeyed power laws [21] (Figure S1), suggesting that a large number of these pathways are influenced by only a small number of CVD drugs and targets, whereas most CVD drugs and targets operate in only a few pathways, which could be the key pathways involved in the therapy of cardiovascular diseases. We applied pathway enrichment analysis [22] to identify these key pathways.

For each of the 120 pathways owning CVD drug targets, we computed the p-values according to the definition of pathway enrichment, respectively. As there are different statistic methods to calculate this value, we adopted three strategies: distinct protein enrichment of CVD drug targets, protein node enrichment of CVD drug targets and CVD drug enrichment (see Methods section for details). The analysis generated 33 pathways (28% of all pathways) with at least one p-value<0.05 (Table S1). The significant differences in the percentage of distinct protein targets and regulating points between all proteins and CVD drug targets on the target-enriched pathways are shown in Figure S2. Enrichment type III pathways are regulated by many more CVD drugs than other categories of drugs (Figure S3). In total, 129 drugs and 103 targets for CVD were associated with these 33 pathways, making up 97% and 79% of all CVD drugs and targets that are related to KEGG pathways, respectively. Therefore, it is reasonable to regard these 33 pathways as key pathways involved in the therapy of cardiovascular diseases. All together, we identified 1,619 proteins involved in these 33 pathways. These proteins could potentially contribute to cardio-protective action of CVD drugs. Hence they were used as candidate protein targets in our study.

### Putative protein targets for AGS-IV

Small-molecule drugs generally function by binding to proteins and/or nucleic acids. Ligand-protein inverse docking is one approach to search for multiple putative protein targets. Base on the 33 key CVD-associated pathways identified by enrichment analysis, we used INVDOCK [16] to identify putative protein targets for AGS-IV.

The candidate 3D dataset included all known information for the 1,619 proteins in the 33 key pathways (total entries: 3,475 PDB). The 3D structure of AGS-IV (Figure S4) was entered into INVDOCK to search for potential interaction. The analysis identified 39 distinct proteins (54 PDB) as putative targets for AGS-IV (Table S2).

**Figure 1:**
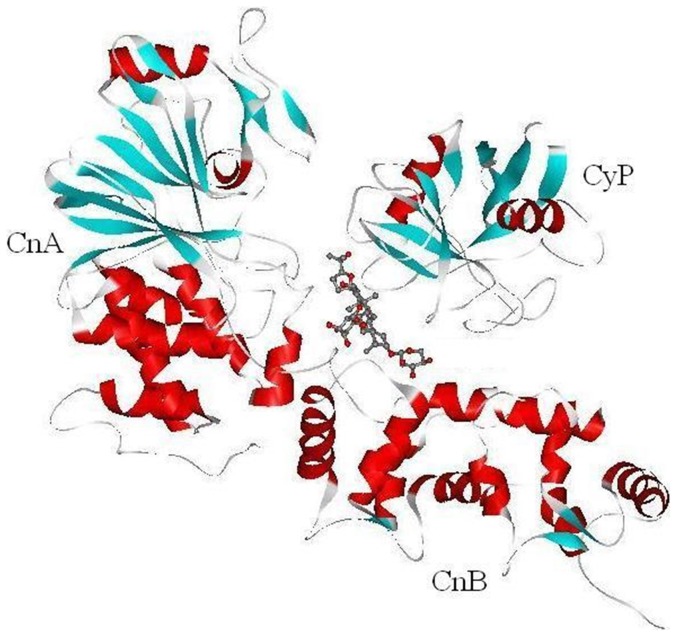
INVDOCK-predicted binding model of AGS-IV molecule (shown in ball and stick model) with cyclophilin (Cyp) and calcineurin (Cn). CnA and CnB are the catalytic subunit and the calcium-binding regulatory subunit of calcineurin, respectively. In the protein secondary structure, red, blue and grey colors represent alpha-helices, beta-sheets and loops, respectively; for the ligand structure, red and grey colors correspond to oxygen and carbon atoms, respectively.

Putative complexes of AGS-IV with calcineurin (CN) and cyclophilin (Cyp) are shown in Figure 1. The immunosuppressive drugs cyclosporin A (CsA) and FK506 bind to Cyp and FK506-binding protein, respectively, forming drug–protein complexes that in turn recruit CN and inhibit its activity [23]. Crystal structures of the Cyp/CsA/CN ternary complex are deposited in PDB database as entries 1mf8 and 1m63 [23], [24]. INVDOCK removes CsA from the complex to generate an active cavity. The program then attempts inverse-docking with the small molecule of interest. Such an analysis indicated that AGS-IV could form a reasonable drug-protein complex with CN and Cyp at the same site as CsA (Figure 1).

The Gene Ontology (GO) terms of the 39 putative protein targets, their implications for CVD therapy, and corresponding references are listed in Table S2. A total of 33 proteins can be classified into 8 functional classes known to be associated with the pathogenesis of CVD (see Table I). The associations of these protein classes with CVD are partially described as follows:

1. An altered balance in the biological action of vasodilator and vasoconstrictor molecules is associated with hypertension.
2. Abnormity in blood coagulation cascade may lead to thrombosis.
3. Calcium ions are key intracellular messengers in the cardiovascular system. Proteins that mediate calcium fluxes in the heart and vascular smooth muscle initiate excitation-contraction coupling.
4. MAP kinase signal transduction pathway has been reported to play a crucial role in many aspects of cardiovascular responses [25].
5. Normal cardiac growth and exercise-induced hypertrophy were found to be regulated by the PI3K/Akt pathway to a high extent [26].
6. Small G proteins have shown to be implicated in regulation of endothelial function, smooth muscle cell contraction, proliferation, and migration, as well as cardiomyocyte hypertrophy [27].
7. Cardiac injury is known to activate innate immune mechanisms initiating an inflammatory reaction, and cardiac repair following myocardial infarction is related with immune response [28].
8. Oxidative stress is associated with many forms of cardiovascular diseases. Increased production of reactive species in vascular tissues may play an important role in hypertension, vascular remodeling after angioplasty, atherosclerosis, myocardial infarction, and ischemic stroke [29].

**Table 1:** Functional classes of putative therapeutic targets of AGS-IV identified by an INVDOCK search of a candidate protein 3D structure data set.

No.	Protein functional class	CVD disease association	Gene ID for putative targets of AGS-IV
1	Regulation of vasoconstriction and vasodilation	Hypertension	*ACE*, *NOS3*
2	Blood coagulation	Thromboembolic diseases	*F2*, *SERPINC1*
3	Calcium ion related	Myocardial ischemia injury [44]	*CALM1*, *CN*, LCK, DAPK1, DAPK2, *MMP1*
4	MAP kinase activity related	Myocardial ischemia injury [47]	*EGFR*, *EGF*, *TGFA*, *FGFR1*, *INSR*, *MAPK12*, *MAPK14*, *MAPK8(JNK)*
5	PI3K/Akt pathway	Hypertrophy [26]	*PIK3CG*, *mTOR*
6	Small G proteins	Cardiomyocyte hypertrophy [27]	*ARF1*, *RAC3*
7	Immune response	Myocardial infarction [28]	*HLA-DRB1*, *HLA-DRA*, *HLA-A*, *BST1*, *IRAK4*
8	Anti-oxidant related	Hypertension, atherosclerosis, myocardial infarction, and ischemic stroke [29]	CYP3A4, ADH5, AKR1C4, GAPDH, *NQO1*, *NCF1*
9	Others	-	KIT, *MME*, OAT, RXRB, MSN, GCK

Targets of approved CVD drugs are underlined. Proteins with reported association with CVD therapy are indicated in italic.

A comprehensive literature search in PubMed showed that 27 of the 33 proteins aforementioned were reported as drug targets or potential targets for CVD treatment (see Table I). All but one of the 27 proteins belong to the 8 functional classes listed above.

### Experimental target validation

CN, ACE and JNK were validated experimentally for two reasons. First, there is ample literature support for important role of these proteins in CVD. Second, these three proteins are readily available.

**Figure 2:**
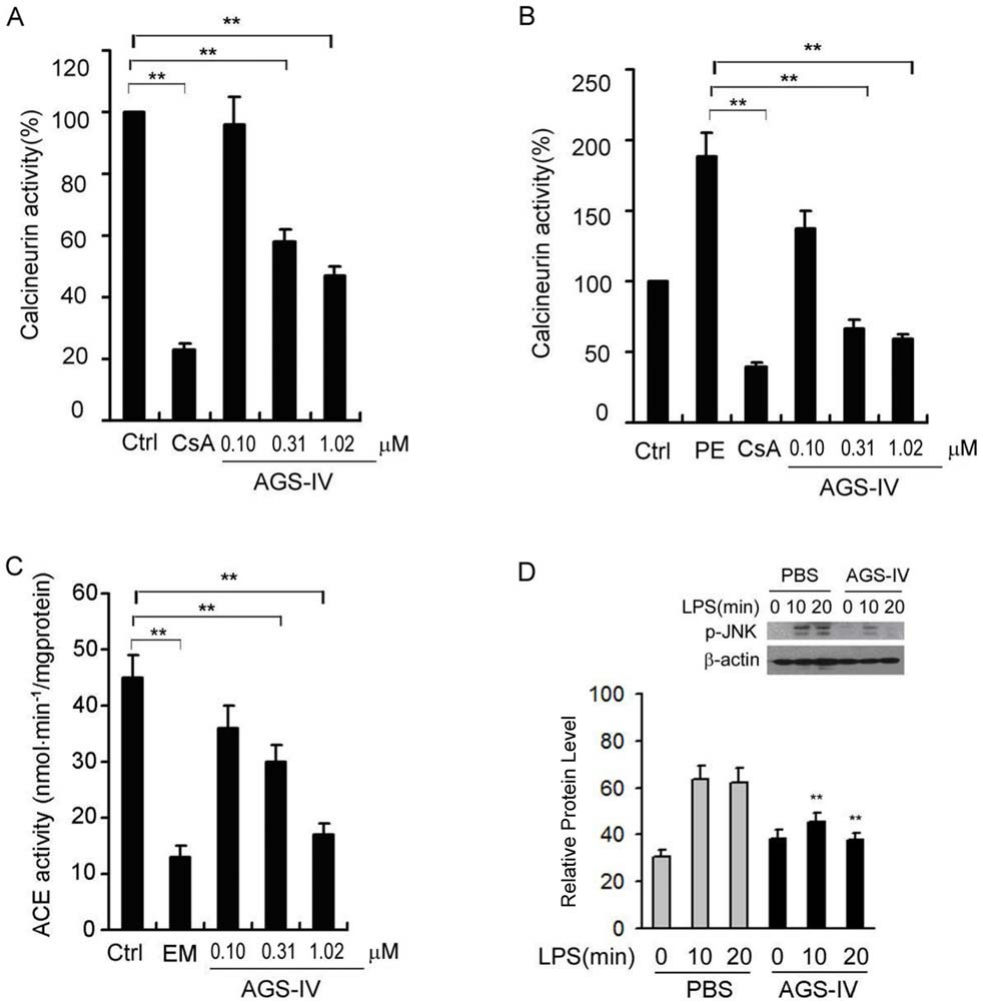
Evaluation of AGS-IV on representative protein targets. (A) Calcineurin activity in VSMCs following a 24-h pretreatment with AGS-IV (0.10∼1.02 µM), while cyclosporin A (CsA) was used as positive control. (B) PE-induced calcineurin activation in VSMCs following a 24-h pretreatment with AGS-IV (0.10∼1.02 µM). (C) ACE activity in HUVECs following a 20-min pretreatment AGS-IV (0.10∼1.02 µM), while enalapril maleate (EM) was used as positive control. (D) AGS-IV (1.02 µM) inhibited LPS-induced activation of JNK for at least 20 min. JNK phosphorylation levels were determined by Western blot, analyzed by Quantity One™ software (BioRad Inc.) and normalized to β-actin.

To investigate the effects of AGS-IV on CN activity, we pretreated the vascular smooth muscle cells (VSMCs) with AGS-IV (0.10∼1.02 µM) for 24 h. As mentioned earlier, INVDOCK predicted that AGS-IV binds to CN in the same pattern as CsA does. Hence CsA (80 µg/L) was added as positive control. Then, CN activities were determined by the CN activity assay kit. Our results indicate that AGS-IV inhibited CN activity with an IC_50_ of 403 nM (Figure 2A). The IC_50_ of CsA, the reference CN inhibitor, is 8.3 nM [30]. In addition, it was reported that phenylephrine (PE) is a stimulator for Ca^2+^ oscillations and CN activation [31]. Thus we determined the effect of AGS-IV on PE-induced CN activation. As can be seen, the increase in CN activity induced by PE (10 µmol/L) for 24 h was inhibited by a 24-h pretreatment with AGS-IV (0.10∼1.02 µM) in a concentration-dependent manner (Figure 2B).

To validate the inhibition of ACE by AGS-IV, we incubated cultured endothelial cells from human umbilical veins (HUVEC) with AGS-IV. Considering our result that AGS-IV docks into the same binding pocket of ACE as ACE inhibitors captopril and enalaprilat [32], we added enalapril maleate (EM, 80 µg/L) as the positive control. After incubation with AGS-IV (80∼800 µg/L) for 20 min, a significant and concentration-dependent inhibition of ACE activity in HUVEC was detected (Figure 2C). Our experiment suggests that AGS-IV inhibited ACE with an IC_50_ of 268 nM. IC_50_ of the reference drug enalapril is 1.94 nM [33].

Inflammation plays a critical role in CVD. Toll-like receptor (TLR) signaling is involved in the pathological process of inflammation in vascular smooth muscle cells (VSMCs) [34]. The expression of certain bacterially responsive TLRs in VSMCs promotes a proinflammatory and proliferative phenotype in which MAPK and NFκB play central roles [34]. To test the effect of AGS-IV on JNK (MAPK8) activity, we pretreated VSMCs with AGS-IV, then used lipopolysaccharide (LPS) to trigger TLR-4 response and determined the resulting level of JNK phosphorylation. As shown in Figure 2D, AGS-IV (1.02 µM) significantly decreased JNK activity at 10 and 20 minutes after LPS challenge.

### The protective effect of AGS-IV on adriamycin-induced injury of cardiomyocytes

Pathological cardiac hypertrophy is a response to a variety of primary conditions such as hypertension, myocardial hypoxia, ischemia and infarction, and is associated with heart failure, arrhythmia and increased risk of heart attack. Previous studies indicated that blocking CN could alleviate cardiac hypertrophy [35]. Based on the finding that AGS-IV could inhibit CN, we next examined whether AGS-IV could alleviate adriamycin-induced injury in cardiomyocytes.

**Figure 3:**
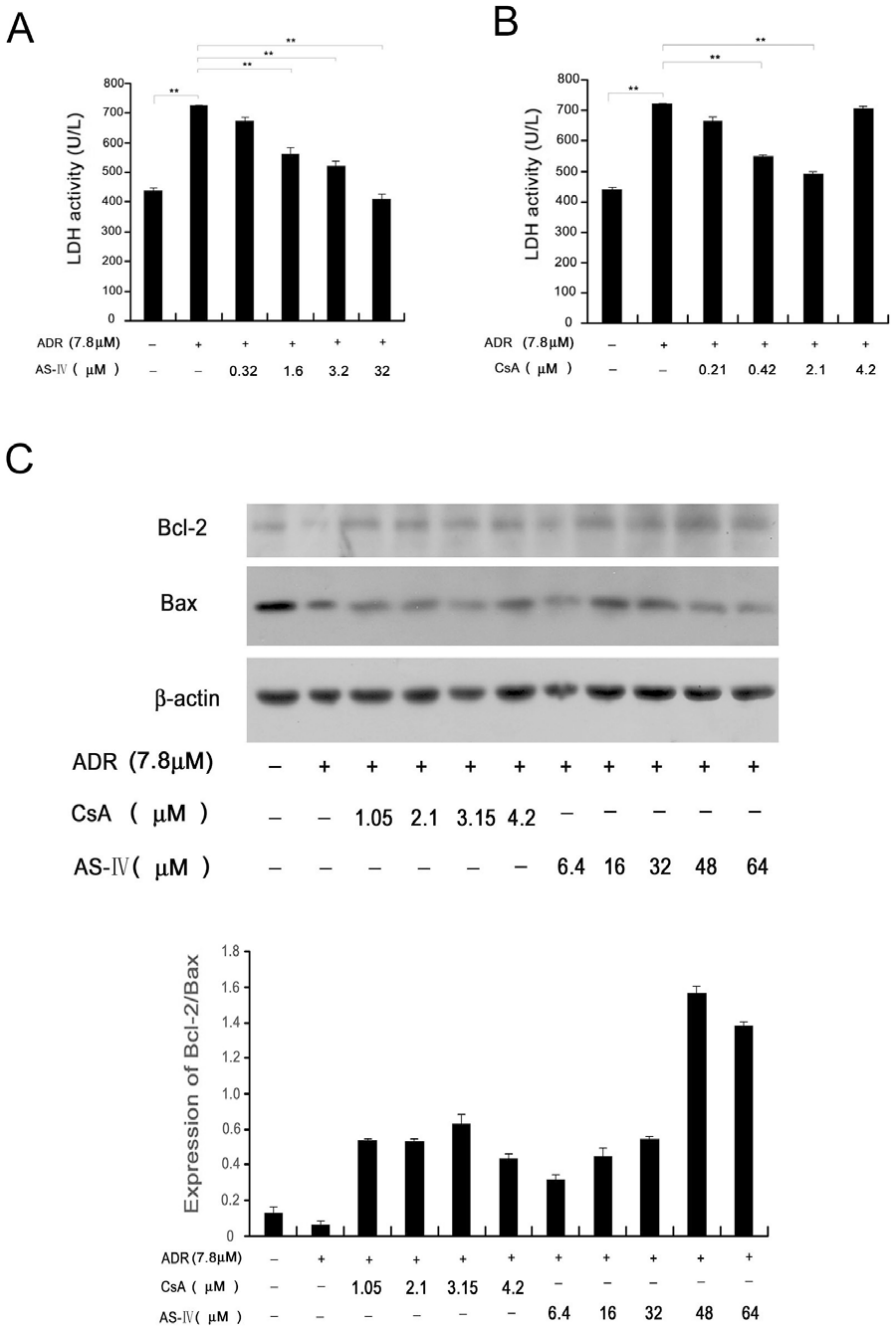
The protective effect of AGS-IV in adriamycin-induced injury of cardiomyocytes compared with CN inhibitor CsA. (A) Activity of LDH in culture supernatant of the ADR-injured cardiomyocytes treated by AGS-IV (0.32∼32 µM). (B) Activity of LDH in culture supernatant of the ADR-injured cardiomyocytes treated by CsA (0.21∼4.2 µM). Data are shown as mean±SD (n = 4) (**P<0.01). (C) The effects of CsA and AS-IV on the expressions of apoptotic proteins. The expression of BCL-2 and Bax in ADR-injured cardiomyocytes treated by CsA or AGS-IV, the effects of CsA and AS-IV on the Bcl-2/Bax level. All data are mean±SD (n = 3).

Lactate dehydrogenase(LDH), an enzyme existed in all cells, could be used as an index of cytotoxic effect, for releasing into the culture supernatant after the injury. We determined the activity of LDH leaking into the medium from ADR-injuried cardiomyocytes treated by AGS-IV or CsA respectively. Our experiments suggested that AGS-IV and CsA inhibited ADR-induced release of lactate dehydrogenase (LDH) from cultured cells to the medium in a concentration-dependent manner. The EC_50_ for CsA and AGS-IV was 365 nM (Figure 3A) and 1.36 µM (Figure 3B), respectively. At the highest concentration of 5 µg/ml, CsA produced some injury in our experiment.

Bcl-2 exerts a survival function in response to a wide range of apoptotic stimuli by inhibiting mitochondrial cytochrome c release; whereas Bax is a key component of apoptosis induction via mitochondrial stress. The Bcl-2/Bax ratio is commonly used to measure the impact of drugs on the apoptotic process. An increase in the ratio suggests a protective effect against apoptosis [36]–[38]. We used Western blot analysis to detect the effects of AGS-IV and CsA on apoptotic proteins. As shown in Figure 3C, both CsA and AGS-IV increased the Bcl-2/Bax ratio in ADR-injured cardiomyocytes, indicating their protective effects against apoptosis. Interestingly, the Bcl-2/Bax ratio decreased at high dose of CsA (4.2 µM) and increased significantly at high dose of AGS-IV (48 and 64 µM) respectively. This phenomenon suggests that AGS-IV has stronger protective effects on ADR-induced apoptosis and weaker side-effects.

In the last section we verified the direct inhibition of AGS-IV on CVD associated active proteins CN, ACE and JNK, here we examined the protective function of AGS-IV on ADR-induced injury in cardiomyocytes. We referred to these two experiments as protein and cellular level experiments, respectively. In Table II we compared the action of AGS-IV and its positive contrasts at the two different levels. The affinity of AGS-IV was 50- and 140-fold lower for CN and ACE than the reference drugs (CsA and Enalapril), respectively. However, at the whole cell level, the inhibitory effect of AGS-IV on ADR-induced LDH release was only 3.7 fold less potent than CsA. This phenomenon suggests a much stronger activity of AGS-IV at the cell level than that at the protein level.

**Table 2:** Potency of AGS-IV against CVD-associated targets.

Level of experiment	Experiment design	AGS-IV	Positive control	AGS-IV/Control IC50 (EC50) Ratio
Protein level	In vitro CN inhibition	IC50 = 403 nM	CN inhibitor cyclosporin A IC50 = 8.3 nM [30]	48.6
	In vitro ACE inhibition	IC50 = 268 nM	ACE inhibitor Enalapril IC50 = 1.94 nM [33]	138.1
Cellular level	In vitro inhibition of ADR-induced increase of LDH activity	EC50 = 1.357 µM	Cyclosporin A 0.365 µM	3.7

### Protein-protein association network of putative targets of AGS-IV

**Figure 4:**
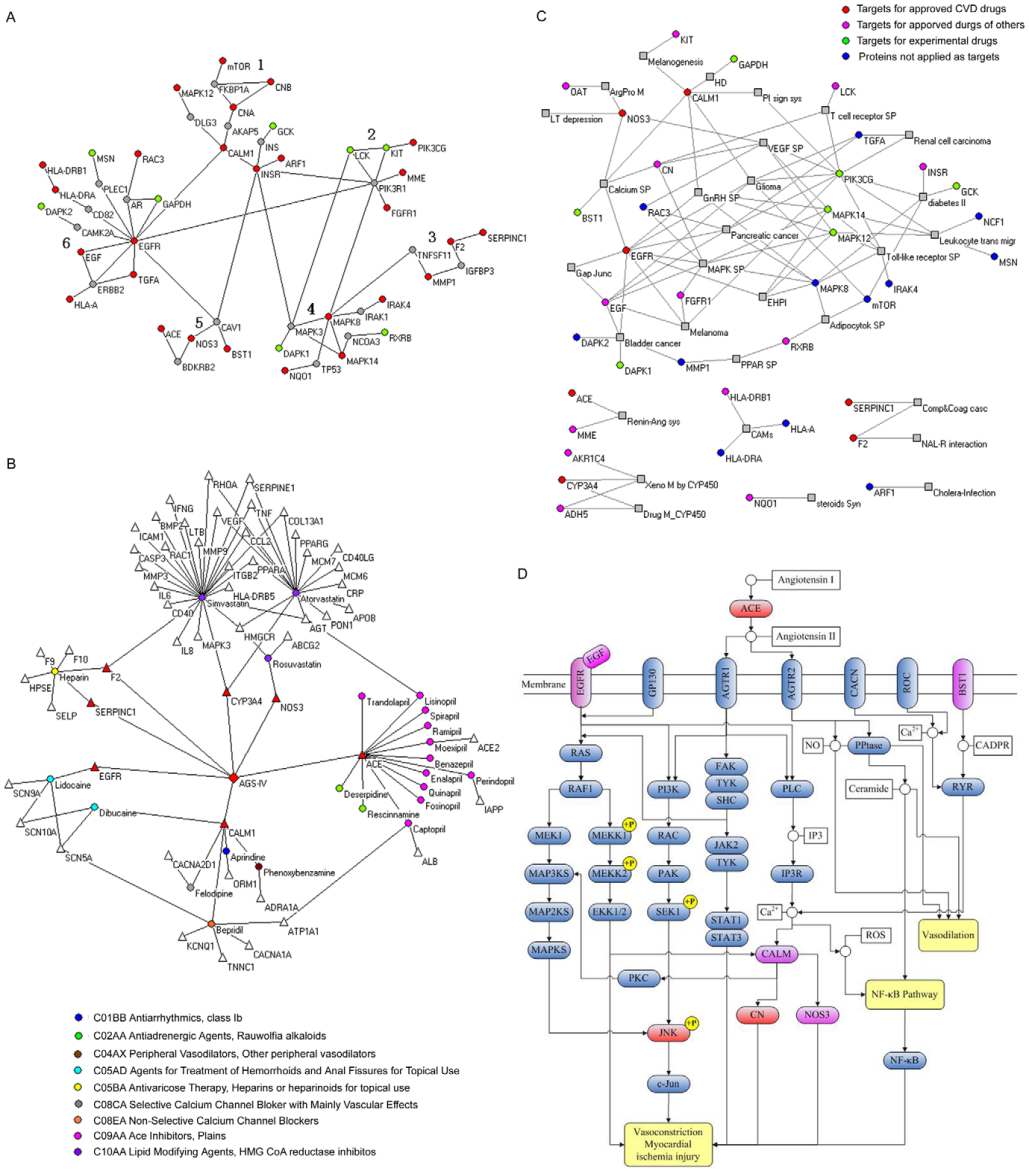
Network analysis of the efficacy of AGS-IV on myocardial injuries. (A) Constructed minimum protein-protein interaction network between targets of AGS-IV. Red and green nodes denote putative targets of AGS-IV. Red nodes have been reported in the literature as drug targets or potential targets for CVD therapy; green nodes are predicted by the INVDOCK but with no literature support. Grey nodes are proteins that link the targets together. The network was decomposed into topological compact modules, labeled with numbers, by a simulated annealing algorithm. (B) Drug-target network for cardiovascular drugs related to AGS-IV. Circles and triangles represent drugs and targets, respectively. AGS-IV and its putative direct targets are highlighted in red. Nodes for other drugs are colored according to their ATC codes at the 4^th^ level (ATC codes are codes in the Anatomical Therapeutic Chemical (ATC) Classification System used for the classification of drugs, which is controlled by the WHO Collaborating Centre for Drug Statistics Methodology (WHOCC). Drugs are classified into groups at 5 different levels in this coding system, in which the 4^th^ level of the code indicates the chemical/therapeutic/pharmacological subgroup). (C) Target-pathway network for putative targets of AGS-IV. Circles and squares represent targets and pathways, respectively. Target nodes are colored according to Drugbank drug classes. Known targets of approved CVD drugs are highlighted in red. (D) Pathway associated with myocardial ischemia injury. This pathway was constructed by integrating pathways reported to be involved in the process of myocardial ischemia injury, including the calcium signaling, MAPK, JAK-STAT and NF-KB pathway. Red and pink boxes indicate putative target proteins of AGS-IV; targets in red were experimentally validated in this study.

To understand the relationship among the putative targets of AGS-IV, we mapped the 39 proteins onto the protein-protein interaction network of the human genome. We successfully identified 37 proteins and found that 34 of them can be linked into one sub-network either through direct interactions or with only one intermediate protein, suggesting that most of the targets are located in the neighborhood of each other in the human protein network. The proximity of proteins in the interactome indicates that they share common functions. To identify the functions of this network between targets, we next applied a simulated annealing algorithm [39] to partition it into six topologically compact modules (Figure 4A). In 4 of the modules, the targets are organized around a common hub protein, such as INSR (insulin receptor) in Module 1, PIK3R1 (PI3-kinase) in Module 2, MAPK8 (c-Jun N-terminal kinase 1, JNK) in Module 4, and EGFR (epidermal growth factor receptor) in Module 6. In some instances, AGS-IV modulates the hubs directly and exerts its influence on the others by acting on their surrounding neighbors. Previous studies on the effect of multi-target attacks in a network-based model suggested that weak inhibition of multiple targets could be more efficient than potent inhibition of a single hub target [15], [40]. We can also see that proteins grouped together tend to be correlated in functionality or to participate in the same biological processes. Specifically, proteins in Module 1 are implicated in calcium signaling and the regulation of glucose metabolism by insulin. Module 2 includes two PI3-kinases, one of which is a hub (PIK3R1) that connects to the PI3K pathway, which in turn is associated with cardiac growth and hypertrophy [25]. Module 3 is an antithrombotic cluster. The proteins in Module 5 are implicated in the regulation of vasoconstriction and vasodilation. The two larger modules, Module 4 and Module 6, are associated with anti-oxidative and anti-inflammatory functions, respectively. Overall, this network suggests that AGS-IV produce its beneficial effects on the cardiovascular system by multiple mechanisms, including anti-oxidative, anti-inflammatory, blocking calcium flux and immune regulation along with the effects on cardiac growth, vasoconstriction and vasodilation. Thus, the organization of the target protein interaction network exhibits a modular cooperative mode and may result in synergistic therapeutic action.

### Drug-target network for AGS-IV and FDA-approved CVD drugs

It would be interesting to bridge AGS-IV and existing FDA-approved CVD drugs via their common drug targets. This is expected to provide alternative insights for deducing the therapeutic mechanism of AGS-IV. Among the 39 putative targets of AGS-IV identified in this study, seven are known targets of approved CVD drugs. We searched the DrugBank database and found 23 approved CVD drugs that share common targets with AGS-IV. We extracted all of the targets of these 23 drugs and used this information to construct a drug-target network (Figure 4B). The anticoagulant heparin shares two targets with AGS-IV (Fig. 4B): F2 (prothrombin) and SERPINC1 (antithrombin-III), suggesting that AGS-IV may also have antithrombotic function. Arterial thrombosis is a major cause of stroke and myocardial infarction. *Astragalus membranaceus* has been widely used in TCM for stroke patients, and a previous clinical investigation suggested that *Astragalus membranaceus* may be beneficial for the treatment of acute cerebral infarction [41]. Moreover, our earlier *in vivo* experiments suggested that AGS-IV could significantly reduce the volume of heart and brain infarctions [4], [7]. The result from the drug-target network suggests that binding with F2 and SERPINC1 contributes to the antithrombotic function of AGS-IV.

ACE inhibitors are widely used in the treatment of cardiovascular diseases, including congestive heart failure, coronary artery disease and hypertension. The drug-target network for AGS-IV also includes a group of ACE inhibitors. Based on these results, we speculate that the cardioprotective effect of AGS-IV may be partially due to its inhibition of ACE. By blocking ACE, ACE inhibitors decrease the level of angiotensin II, a vasoconstrictor and a negative feedback mediator of renin activity. Our earlier experimental study found that AGS-IV dilates aortic vessels through angiotensin II inhibition [6]. The drug-target network analysis suggests that inhibition of ACE could also contribute to the vessel dilatation function of AGS-IV. Likewise, binding with calmodulin (CALM1) may allow AGS-IV to act as a calmodulin-blocking agent, a class of approved drugs for the treatment of CVD by blocking calcium flux.

The network we constructed for AGS-IV includes three statin drugs (ATC code C10AA). However, the primary target of statins (HMG-CoA reductase, HMGCR) is not a target for AGS-IV. Two local anesthetics, lidocaine and dibucaine (ATC code C05AD), share two common targets with AGS-IV: EGFR and CALM1. But the targets are apparently not relevant to the therapeutic action of AGS-IV.

### Target-pathway network for key pathways associated with AGS-IV therapy for CVD

To get an overall picture of the interplay between AGS-IV's targets and CVD therapy-associated pathways, we mapped the 39 putative protein targets of AGS-IV onto the 33 key pathways associated with CVD therapy and found that they appeared in 30 out of the 33 key pathways. A target-pathway network for these targets and pathways is illustrated in Figure 4C. This network consists of one large, highly connected cluster and six small, isolated clusters. About half of the targets appear in multiple pathways. These proteins may mediate the interactions and cross-talk between different pathways, indicating that AGS-IV may intervene in more pathways related to CVD via limited number of targets. Several pathways are modulated by AGS-IV through multiple target proteins. The pathways that incorporate multiple targets, such as the MAPK, VEGF and calcium signaling pathways could be the primary pathways by which AGS-IV produces its therapeutic effects on CVD. In fact, these pathways have been reported as suitable target pathways for cardiovascular disease therapies [42], [43]. We constructed a myocardial ischemia associated pathway (Figure 4D) by integrating pathways reported to be involved in the process of myocardial ischemia injury, including the calcium signaling, MAPK, JAK-STAT and NF-KB pathway [44]–[47]. Four targets for approved CVD drugs, ACE, EGFR, CALM and NOS3, are linked with this pathway. AGS-IV targets seven proteins in this pathway, three of which are located upstream of the pathway and four of which are downstream. Among the latter group, AGS-IV targets CALM, CN and JNK. JNK kinase activity is activated by ischemia/reperfusion [47]. Ca^2+^ influx accumulation is implicated in the pathogenesis of ischemic and reperfusion injury [44]. CN is a calcium-dependent protein phosphatase that is activated by binding of Ca^2+^ and CALM to the regulatory and catalytic subunits, respectively [48]. CN activity in the heart is increased upon ischemia [49]. Consistently, inhibiting CALM could alleviate myocardial ischemia reperfusion injury [44]. These facts suggest important roles of JNK, CN and CALM in the therapeutic action of AGS-IV against myocardial ischemia. On the other hand, two of the upstream targets, epidermal growth factor receptor (EGFR) and ACE, have also been proposed as potential targets for myocardial ischemia [47], [50]. A mode of action in which AGS-IV simultaneously regulates multiple proteins related to myocardial ischemia localized both upstream and downstream of the pathway may result in a refined and synergistic modulation effect upon the disease.

## Discussion

Systems biology and network pharmacology are attracting increasing attention in drug discovery. In contrast to the classical strategy of targeting individual proteins, network-based approach considers the overall effects produced by multiple targets, including network robustness [51], pathway bypass, redundancy [51], and crosstalk [52], and has the potential to increase success rate in drug development [53]. Natural compounds tend to affect multiple targets. As a result, network-based approach is particularly useful for drug design using natural compounds as template [54].

The current study conducted a network-level investigation for the therapeutic mechanisms of AGS-IV on CVD. To maintain a reasonable level of reliability without substantially sacrificing statistical power, we focused on the 33 key pathways known to be affected by majority of FDA-approved CVD drugs. We used a flexible ligand-protein inverse docking program, INVDOCK, to pinpoint proteins with which AGS-IV may potentially interact. We are not presently able to identify potential target proteins without an existing 3D structure. Among the 39 potential targets predicted from an analysis of the crystal structures of 1,619 proteins, 69% have been reported in the literature as known or potential targets for CVD drugs. Three proteins (e.g., ACE, CN and JNK) with the strongest literature support were experimentally verified. These results suggest that target prediction based on protein candidates selected from key CVD related pathways is a sound approach in identifying drug targets.

A recent study suggested that both AGS-IV and the ACE inhibitor quinapril could improve cardiac function in a rat model of chronic heart failure [55]. Another study demonstrated protective effects of AGS-IV similar to the beta-adrenergic blocker propranolol in a mouse model of cardiac hypertrophy [2]. Results from the current study demonstrated activity of AGS-IV in specific protein targets, but with much weaker potency in comparison to relevant FDA-approved drugs. For example, IC_50_ of AGS-IV against purified CN and ACE was approximately two orders higher than CsA and enalapril, respectively. Considering the fact that AGS-IV is a naturally-occurring substance, such results are not surprising. At the whole cell level (adriamycin-induced LDH release from cultured cardiomyocytes), however, AGS-IV is only 3.7 fold less potent than CsA. The much stronger activity of AGS-IV at the cell level suggested synergistic action among multiple targets. Our experiments validated the network-based mathematical models, and suggested that partial inhibition of multiple targets can be more efficient than potent inhibition of a single target [15].

Our network analysis suggests that the therapeutic effects of AGS-IV are likely achieved by modulation of a combination of targets in a modular pattern. Of particular interest are target proteins at the crosstalk sites among the multiple pathways that participate in calcium, MAPK, VEGF signaling (e.g., JNK, CN and EGFR). This allows AGS-IV to intervene in multiple pathways involved in CVD via limited number of targets.

The network-based systems biology strategy described in this paper could be an effective approach for elucidating the protein relationships within and between comprehensive pathways in complex diseases and their treatment. In our knowledge, this study is the first to employ a network-based computational approach in systematic search of potential CVD-related drug targets for a natural compound and to illustrate its underlying molecular mechanism based on the various network associations. Also, the multiple mechanisms identified in this study could be valuable for designing new network-based multi-target CVD drugs or for proposing combinations of drug treatment for CVD.

## Materials and Methods

### Data Preparation

#### CVD drugs and therapeutic targets

Data about drugs and targets were downloaded from the DrugBank database [19] in June of 2011. We searched the DrugBank database and extracted all of the FDA approved small molecule drugs for cardiovascular diseases and their corresponding targets (174 drugs and 188 protein targets).

#### Pathway data

Pathway data was downloaded from the FTP service of KEGG [20] (Kyoto Encyclopedia of Genes and Genomes) in June of 2011. The KEGG PATHWAY section is a collection of manually constructed pathway maps representing information on molecular interaction and reaction networks. The “hsa_pathway.list” file in this section includes a list of known pathways occurring in human biological processes and proteins participating in these pathways. As of June of 2011, KEGG included 235 pathways. These pathways are organized hierarchically as shown on the KEGG website.

#### Protein-protein interaction data

Protein-protein interaction data were downloaded from the HPRD database (Human Protein Reference Database, release_9) [56]. The database includes 9673 human proteins and 39,240 interactions manually extracted from the literature based on *in vivo*, *in vitro*, or yeast two-hybrid experiments.

#### Identification of putative protein targets for AGS-IV

INVDOCK software [16] was used to identify putative protein targets for AGS-IV. The 3D structure of AGS-IV was input into the INVDOCK program. The software automatically searched for protein cavities derived from 3D structures of all candidate proteins. An energy value statistically derived from the analysis of a large number of PDB ligand-protein complexes was used as a threshold for screening likely binders [16].A human protein was considered as a putative target of AGS-IV if the molecule could be docked into the protein and the binding satisfied a molecular-mechanics based criterion for chemical complementarity [16].

### Network construction and analysis

#### Construction of protein-protein interaction network between AGS-putative targets

We first constructed a protein-protein interaction network for human genome based on the HPRD data and mapped all putative targets of AGS-IV onto this network. Then we applied the Steiner minimal tree algorithm [57] to identify a minimum sub-network, which included as many putative targets of AGS-IV and as few other proteins as possible. Each target protein of AGS-IV was allowed to interact with other target proteins through no more than one non-target protein.

#### Construction of drug-target network

A drug-target network is defined as a bipartite network for the drug-target associations consisting of two disjoint sets of nodes [58]. One set of nodes corresponds to all drugs under consideration, and the other set corresponds to all the proteins targeted by drugs in the study set. A protein node and a drug node are linked if the protein is targeted by that specific drug according to the DrugBank information.

#### Construction of target-pathway network and drug-pathway network

We first mapped all the protein targets of the drugs under study onto the KEGG pathways [20]. The proteins appearing in any one pathway were considered to participate in that specific pathway. The drugs binding to those target proteins may therefore act on that pathway. All of the pathways and the involved target proteins derived from this search were used to generate a bipartite graph of target-pathway association. A target and pathway were linked if the target appears in that pathway. Likewise, a drug-pathway network was constructed in which a drug and a pathway were linked if at least one target of the drug mapped onto that pathway.

#### Degree distribution of network

In a network, the degree *k* of a node is the number of edges linked to that node. The degree distribution *p(k)* of a network is the frequency of occurrence of nodes with degree *k*, (*k* = 1,2,…). It has been reported that degree distributions of most real world networks including biological networks obey power law. Such distribution suggests that most nodes in the network (corresponding to entities of the system under study) only influence limited number of nodes, while a small number of nodes interact with quite plenty of nodes thus play key roles in the system [59].

#### Pathway enrichment of targets and drugs

We used pathway enrichment analysis [22] to determine whether a pathway is significantly regulated by CVD drugs. We used hypergeometric cumulative distribution [60] to quantitatively measure whether a pathway is more enriched with CVD drug targets (or drugs) than would be expected by chance. A significance level (alpha) with a P-value<0.05 demonstrates a low probability that the CVD drug targets (or drugs) appear in the pathway by chance, *i.e.*, the pathway can be regarded as significantly regulated by CVD drugs. We defined three types of pathway enrichment as follows:

- Type I enrichment: Distinct CVD drug targets are significantly enriched in the pathway.
- Type II enrichment: Regulating points of CVD drug targets are significantly enriched in the pathway. This type of enrichment is defined to identify pathways that are regulated by few distinct protein targets but at many positions, that is, one target appears at multiple positions of the same pathway.
- Type III enrichment: CVD drugs are significantly enriched in the pathway.

#### Network decomposition

We applied the simulated annealing algorithm [39], [61] to break up the protein interaction network between AGS-IV's putative targets. This algorithm identifies topological modules by maximizing the network's modularity metric through an stochastic optimization technique that enables one to find ‘low cost’ configurations without getting trapped in ‘high cost’ local minima, thus may generate a nearly best decomposition of the network. For a given decomposition of a network, the modularity metric is defined as the gap between the fraction of arcs within clusters and the expected fraction of arcs if the arcs are wired with no structural bias [62]:
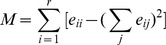
where *r* is the number of clusters, 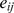 is the fraction of arcs that leads between vertices of cluster *i* and *j*. The maximum modularity metric corresponds to the partition that comprises as many as within-module links and as few as possible inter-module links.

### Experimental tests

#### Drug preparation

Dulbecco's modified Eagle's medium (DMEM) media, fetal bovine serum and 0.25% trypsin-EDTA for the cell culture were purchased from Invitrogen (Carlsbad, CA, USA). AGS-IV (lot no. 030510) was extracted from *Astragalus membranaceus* (Fisch) Bunge to a 99.2% purity (HPLC analysis) and dissolved in DMSO. The calcineurin assay kit was purchased from Nanjing Jiancheng Bioengineering Institute (NJBI) (Nanjing, China). Phenylephrine (PE), cyclosporin A (CsA) and enalapril maleate (EM) were purchased from Sigma-Aldrich (St. Louis, MO, USA). Anti-phospho-JNK mAb, anti-JNK mAb, and their respective horseradish peroxidase-coupled secondary antibodies were purchased from Santa Cruz (Santa Cruz, CA). All other reagents were purchased from Sigma (St. Louis, MO, USA).

#### Culture of VSMC

All research involving animals complied with protocols approved by the Animal Care and Use Committee in Second Military Medical University. The medial layer of the thoracic aorta from 7-day-old Wistar rats was extracted and cultured in DMEM containing 5% FBS at 37°C in a 5% CO_2_ atmosphere. The cells were seeded onto six-well plates or 35-mm dishes and cultured to a near confluent condition. Primary VSMC (<4 passages) were used.

#### Calcineurin enzymatic activity

The activity of calcineurin was determined using a calcineurin activity assay kit as described in the manufacturer's protocol. The RII-phosphopeptide (BioMol) was used as a highly specific substrate for calcineurin. The detection of free inorganic phosphate released from RII by calcineurin was based on the malachite green dye reaction. Reactions were terminated after 30 min, and absorption was determined by UV spectroscopy at 660 nm. The activity was corrected for protein concentration. Calcineurin activity was expressed as percentage relative to the PBS control.

#### ACE activity

The ACE activity of human endothelial cells (HUVECs, purchased from Chinese Institute of Biochemistry and Cell Biology) was determined by the rate of production of hippuric acid from the synthetic tripeptide substrate hippuryl-L-histidyl-L-leucine (HHL) according to previously published methods [63].

#### JNK activity

VSMCs were plated in 6-well plates and incubated with either AGS-IV (800 µg/L) or PBS for 30 min. After stimulation with 100 ng/L LPS for 10 or 20 min, the cells were lysed and phosphorylated JNK was detected by Western blotting. JNK activation was normalized to β-actin.

#### Western Blot Analysis

Cells were lysed with M-PER protein extraction reagent (Pierce, Rockford, IL) supplemented with protease inhibitor cocktail and protein concentrations in the extracts were measured by a bicinchoninic acid (BCA) assay (Pierce). Forty micrograms of the protein was used either for immunoprecipitation or subjected to sodium dodecyl sulfate–polyacrylamide gel electrophoresis (SDS-PAGE) and transferred onto nitrocellulose membranes. The β-actin content of each sample was determined to demonstrate equal protein loading.

#### Primary culture of cardiomyocytes from neonatal mice

We applied a well-documented improved anchorage velocity-dependent separation method to separate and purify the cardiomyocytes [64]–[66].

#### Drug treatment

Cardiomyocytes were seeded into a 12-well culture plate at a density of 1∼5×10^5^ cells/ml. Twenty four h later, cardiomyocytes were incubated with different concentrations of CsA (0.25, 0.5, 2.5, 5 µg/ml) or AS-IV (0.25, 1.25, 2.5, 25 µg/ml) for 1 h, then stimulated with adriamycin (ADR) (4.5 µg/ml) for 24 h.

#### LDH activity analysis

The activity of LDH in the culture supernatant of drug-treated cardiomyocytes was determined using an LDH activity assay kit (JianCheng BioTech, Nanjing, China).

## Supporting Information

Figure S1 **Log-log plots for degree distribution of pathway nodes in target-pathway network (a) and drug-pathway network (b) for FDA-approved small molecule CVD drugs.** Distribution of pathway nodes in both of the networks obeyed power laws.
(TIF)

Figure S2 **Comparison of percentage of distinct protein targets and regulating points for cardiovascular targets significantly enriched pathways (P<0.05).** Pathways permuted decreasingly according to percentage. (a) Type I enrichment pathways: enrichment of distinct CVD drug targets (b) Type II enrichment pathways: enrichment of CVD drug target nodes. Pathway names are listed in Table S1.
(TIF)

Figure S3 **Comparison of percentage of FDA-approved drugs acting on cardiovascular drugs significantly enriched pathways (P<0.05).** Pathways permuted decreasingly according to percentage. Pathway names are listed in Table S1.
(TIF)

Figure S4 **Chemical structure and 3D molecular structure of Astragaloside IV.** (a) Chemical structure of AGS-IV. (b) 3D molecular structure of AGS-IV. Grey, red, and white colors represent carbon, oxygen and hydrogen atom, respectively.
(TIF)

Table S1 List of 33 KEGG pathways that are significantly regulated by CVD drugs.
(PDF)

Table S2 **Putative therapeutic targets of AGS-IV identified from INVDOCK search of candidate protein 3D structure data set.** PDB ID is the identification number for a protein in the PDB. Target Class: **AC**: target for approved CVD drugs; **A**: target for approved drugs of other classes; **E**: target for experimental drugs; **U**: protein not applied as target. The properties of proteins with no references were obtained from the Uniprot database. Proteins that have been reported in the literature as associated with CVD therapy are indicated in bold.
(PDF)

## References

[1] Wang H, He K, Ye J (1987) Chemical constituents of Astragalus Mongholicus. Zhong Cao Yao 18: 5–7.

[2] Xu X, Ji H, Gu S, Huang Q, Chen Y (2007) Protective effects of Astragaloside IV on isoproterenol-induced cardiac hypertrophy in mice. J Chin Pharm Univ 38: 451–455.

[3] Wang J, Liu JH, Yao P, Chen YL, Xu C, et al (2007) Protective effect of Astragaloside IV on isoproterenol-induced myocardial injury. J Shanghai Jiaotong Univ(Medical Science) 27: 384–386.

[4] Zhang WD, Chen H, Zhang C, Liu RH, Li H, et al (2006) Astragaloside IV from Astragalus membranaceus shows cardioprotection during myocardial ischemia in vivo and in vitro. Planta Med 72: 4–8. 16450288 10.1055/s-2005-873126

[5] Hu JY, Han J, Chu Zg, Song Hp, Zhang Dx, et al (2009) Astragaloside IV attenuates hypoxia-induced cardiomyocyte damage in rats by upregulating superoxide dismutase-1 levels. Clin Exp Pharmacol Physiol 36: 351–357. 18986331 10.1111/j.1440-1681.2008.05059.x

[6] Zhang WD, Zhang C, Wang XH, Gao PJ, Zhu DL, et al (2006) Astragaloside IV dilates aortic vessels from normal and spontaneously hypertensive rats through endothelium-dependent and endothelium-independent ways. Planta Med 72: 621–626. 16732512 10.1055/s-2006-931572

[7] Luo YM, Qin Z, Hong Z, Zhang XM, Ding D, et al (2004) Astragaloside IV protects against ischemic brain injury in a murine model of transient focal ischemia. Neurosci Lett 363: 218–223. 15182947 10.1016/j.neulet.2004.03.036

[8] Zhang C, Wang XH, Zhong MF, Liu RH, Li HL, et al (2007) Mechanisms underlying vasorelaxant action of Astragaloside IV in isolated rat aortic rings. Clin Exp Pharmacol Physiol 34: 387–392. 17439405 10.1111/j.1440-1681.2007.04564.x

[9] Zhang WJ, Hufnagl P, Binder BR, Wojta J (2003) Antiinflammatory activity of Astragaloside IV is mediated by inhibition of NF-kappaB activation and adhesion molecule expression. Thromb Haemost 90: 904–914. 14597987 10.1160/TH03-03-0136

[10] Jones S, Zhang X, Parsons DW, Lin JCH, Leary RJ, et al (2008) Core signaling pathways in human pancreatic cancers revealed by global genomic analyses. Science 321: 1801–1806. 18772397 10.1126/science.1164368 PMC2848990

[11] Pujana MA, Han JDJ, Starita LM, Stevens KN, Tewari M, et al (2007) Network modeling links breast cancer susceptibility and centrosome dysfunction. Nat Genet 39: 1338–1349. 17922014 10.1038/ng.2007.2

[12] Zhao J, Chen J, Yang T-H, Holme P (2012) Insights into the pathogenesis of axial spondyloarthropathy from network and pathway analysis. BMC Systems Biology 6: S4. 10.1186/1752-0509-6-S1-S4 PMC3403611 23046677

[13] Zhao J, Yang T-H, Huang Y, Holme P (2011) Ranking candidate disease genes from gene expression and protein interaction: a Katz-centrality based approach. PLOS ONE 6: e24306. 21912686 10.1371/journal.pone.0024306 PMC3166320

[14] Hopkins AL (2008) Network pharmacology: the next paradigm in drug discovery. Nat Chem Biol 4: 682–690. 18936753 10.1038/nchembio.118

[15] Csermely P, Agoston V, Pongor S (2005) The efficiency of multi-target drugs: the network approach might help drug design. Trends Pharmacol Sci 26: 178–182. 15808341 10.1016/j.tips.2005.02.007

[16] Chen YZ, Zhi DG (2001) Ligand-protein inverse docking and its potential use in the computer search of protein targets of a small molecule. Proteins: Struct, Funct, Genet 43: 217–226. 11276090 10.1002/1097-0134(20010501)43:2<217::aid-prot1032>3.0.co;2-g

[17] Liew CC, Dzau VJ (2004) Molecular genetics and genomics of heart failure. Nat Rev Genet 5: 811–825. 15520791 10.1038/nrg1470

[18] Epstein J, Rader D, Parmacek M (2002) Perspective: cardiovascular disease in the postgenomic era–lessons learned and challenges ahead. Endocrinology 143: 2045–2050. 12021168 10.1210/endo.143.6.8910

[19] Wishart DS, Knox C, Guo AC, Shrivastava S, Hassanali M, et al (2006) DrugBank: a comprehensive resource for in silico drug discovery and exploration. Nucleic Acids Res 34: D668–672. 16381955 10.1093/nar/gkj067 PMC1347430

[20] Kanehisa M, Goto S (2000) KEGG: Kyoto encyclopedia of genes and genomes. Nucleic Acids Res 28: 27–30. 10592173 10.1093/nar/28.1.27 PMC102409

[21] Albert R (2005) Scale-free networks in cell biology. J Cell Sci 118: 4947–4957. 16254242 10.1242/jcs.02714

[22] Curtis RK, Oresic M, Vidal-Puig A (2005) Pathways to the analysis of microarray data. Trends Biotechnol 23: 429–435. 15950303 10.1016/j.tibtech.2005.05.011

[23] Jin L, Harrison SC (2002) Crystal structure of human calcineurin complexed with cyclosporin A and human cyclophilin. Proc Natl Acad Sci USA 99: 13522–13526. 12357034 10.1073/pnas.212504399 PMC129706

[24] Huai Q, Kim HY, Liu Y, Zhao Y, Mondragon A, et al (2002) Crystal structure of calcineurin-cyclophilin-cyclosporin shows common but distinct recognition of immunophilin-drug complexes. Proc Natl Acad Sci USA 99: 12037–12042. 12218175 10.1073/pnas.192206699 PMC129394

[25] Pandya N, Santani D, Jain S (2005) Role of mitogen-activated protein (MAP) kinases in cardiovascular diseases. Cardiovasc Drug Rev 23: 247–254. 16252017 10.1111/j.1527-3466.2005.tb00169.x

[26] Luedde M, Katus HA, Frey N (2006) Novel molecular targets in the treatment of cardiac hypertrophy. Recent Pat Cardiovasc Drug Discov 1: 1–20. 18221071 10.2174/157489006775244290

[27] Barandier C, Ming XF, Yang ZH (2003) Small G proteins as novel therapeutic targets in cardiovascular medicine. News Physiol Sci 18: 18–22. 12531927 10.1152/nips.01407.2002

[28] Frangogiannis NG (2008) The immune system and cardiac repair. Pharmacol Res 58: 88–111. 18620057 10.1016/j.phrs.2008.06.007 PMC2642482

[29] Wattanapitayakul SK, Bauer JA (2001) Oxidative pathways in cardiovascular disease: roles, mechanisms, and therapeutic implications. Pharmacol Ther 89: 187–206. 11316520 10.1016/s0163-7258(00)00114-5

[30] Kung L, Batiuk T, Palomo-Pinon S, Noujaim J, Helms L, et al (2001) Tissue distribution of calcineurin and its sensitivity to inhibition by cyclosporine. Am J Transplant 1: 325–333. 12099376 10.1034/j.1600-6143.2001.10407.x

[31] Molkentin JD, Lu J-R, Antos CL, Markham B, Richardson J, et al (1998) A Calcineurin-Dependent Transcriptional Pathway for Cardiac Hypertrophy. Cell 93: 215–228. 9568714 10.1016/s0092-8674(00)81573-1 PMC4459646

[32] Natesh R, Schwager SLU, Evans HR, Sturrock ED, Acharya KR (2004) Structural Details on the Binding of Antihypertensive Drugs Captopril and Enalaprilat to Human Testicular Angiotensin I-Converting Enzyme. Biochemistry 43: 8718–8724. 15236580 10.1021/bi049480n

[33] Ceconi C, Francolini G, Olivares A, Comini L, Bachetti T, et al (2007) Angiotensin-converting enzyme (ACE) inhibitors have different selectivity for bradykinin binding sites of human somatic ACE. Eur J Pharmacol 577: 1–6. 17716647 10.1016/j.ejphar.2007.07.061

[34] Yang X, Coriolan D, Murthy V, Schultz K, Golenbock D, et al (2005) Proinflammatory phenotype of vascular smooth muscle cells: role of efficient Toll-like receptor 4 signaling. Am J Physiol Heart Circ Physiol 289: H1069–1076. 15863460 10.1152/ajpheart.00143.2005

[35] Sussman MA, Lim HW, Gude N, Taigen T, Olson EN, et al (1998) Prevention of cardiac hypertrophy in mice by calcineurin inhibition. Science 281: 1690–1693. 9733519 10.1126/science.281.5383.1690

[36] Kalyanaraman B, Joseph J, Kalivendi S, Wang S, Konorev E, et al (2002) Doxorubicin-induced apoptosis: implications in cardiotoxicity. Mol Cell Biochem 234–235: 119–124. 12162424

[37] Narula J, Haider N, Virmani R, DiSalvo T, Kolodgie F, et al (1996) Apoptosis in myocytes in end-stage heart failure. N Engl J Med 335: 1182–1189. 8815940 10.1056/NEJM199610173351603

[38] Crow MT, Mani K, Nam YJ, Kitsis RN (2004) The mitochondrial death pathway and cardiac myocyte apoptosis. Circ Res 95: 957–970. 15539639 10.1161/01.RES.0000148632.35500.d9

[39] Guimera R, Amaral LAN (2005) Cartography of complex networks: modules and universal roles. J Stat Mech: Theory Exp P02001. 10.1088/1742-5468/2005/02/P02001 PMC2151742 18159217

[40] Ágoston V, Csermely P, Pongor S (2005) Multiple, weak hits confuse complex systems:A transcriptional regulatory network as an example. Phys Rev E 71: 051909. 10.1103/PhysRevE.71.051909 16089573

[41] Cai YH, Qin Z, Yao QL (1994) A prospective, pilot study of Astragalus membranaceus in the treatment of acute cerebral infarction. J ClinNeurol 7: 216–218.

[42] Wheeler-Jones CPD (2005) Cell signalling in the cardiovascular system: an overview. Heart 91: 1366–1374. 16162635 10.1136/hrt.2005.072280 PMC1769154

[43] Rajalakshmi DC, Gopalakrishnan ARK, Kartha CC (2008) VEGF signaling: a therapeutic target for cardiovascular disease. . Adv Biochem in Health and Disease 3.

[44] Kimura Y, Engelman RM, Rousou J, Flack J, Iyengar J, et al (1992) Moderation of myocardial ischemia reperfusion injury by calcium channel and calmodulin receptor inhibition. Heart Vessels 7: 189–195. 1336774 10.1007/BF01744603

[45] Yang J, Jiang H, Yang J, Ding J-W, Chen L-H, et al (2009) Valsartan preconditioning protects against myocardial ischemia–reperfusion injury through TLR4/NF-kB signaling pathway. Mol Cell Biochem 330: 39–46. 19370315 10.1007/s11010-009-0098-1

[46] Ananthakrishnan R, Hallam K, Li Q, Ramasamy R (2005) JAK-STAT pathway in cardiac ischemic stress. Vasc Pharmacol 43: 353–356. 10.1016/j.vph.2005.08.020 16260187

[47] Armstrong SC (2004) Protein kinase activation and myocardial ischemia/reperfusion injury. Cardiovasc Res 61: 427–436. 14962474 10.1016/j.cardiores.2003.09.031

[48] Stemmer PM, Klee CB (1994) Dual calcium Ion regulation of calcineurin by calmodulin and calcineurin B. Biochemistry 33: 6859–6866. 8204620 10.1021/bi00188a015

[49] Lakshmikuttyamma A, Selvakumar P, Sharma AR, Sharma RK (2005) Involvement of calcineurin in ischemic myocardial damage. Int J Angiology 14: 1–6.

[50] Dai W, Kloner R (2011) Potential role of renin-angiotensin system blockade for preventing myocardial ischemia/reperfusion injury and remodeling after myocardial infarction. Postgrad Med 123: 49–55. 21474893 10.3810/pgm.2011.03.2263

[51] Kitano H (2004) Biological robustness. Nat Rev Genet 5: 826–837. 15520792 10.1038/nrg1471

[52] Peng X, Karna P, Cao Z, Jiang B, Zhou M, et al (2006) Cross-talk between epidermal growth factor receptor and HIF-1 signal pathways increases resistance to apoptosis by upregulating survivin gene expression. J Biol Chem M603414200. 10.1074/jbc.M603414200 PMC3132567 16847054

[53] Jia J, Zhu F, Ma X, Cao ZW, Li YX, et al (2009) Mechanisms of drug combinations: interaction and network perspectives. Nat Rev Drug Discov 8: 111–128. 19180105 10.1038/nrd2683

[54] Zhao J, Jiang P, Zhang WD (2010) Molecular networks for the study of TCM pharmacology. Briefings Bioinf 11: 417–430. 10.1093/bib/bbp063 20038567

[55] Zhao Z, Wang W, Wang F, Zhao K, Han Y, et al (2009) Effects of Astragaloside IV on heart failure in rats. Chin Med 4: 6. 19338675 10.1186/1749-8546-4-6 PMC2674594

[56] Keshava Prasad TS, Goel R, Kandasamy K, Keerthikumar S, Kumar S, et al (2009) Human Protein Reference Database–2009 update. Nucleic Acids Res 37: D767–772. 18988627 10.1093/nar/gkn892 PMC2686490

[57] Klein P, Ravi R (1995) A nearly best-possible approximation algorithm for node-weighted steiner trees. J Algorithms 19: 104–114.

[58] Yıldırım MA, Goh K-I, Cusick ME, Barabási A-L, Vidal M (2007) Drug-target network. Nat Biotech 25: 1119–1126. 10.1038/nbt1338 17921997

[59] Barabasi AL, Albert R (1999) Emergence of scaling in random networks. Science 286: 509–512. 10521342 10.1126/science.286.5439.509

[60] Zhao J, Ding G-H, Tao L, Yu H, Yu ZH, et al (2007) Modular co-evolution of metabolic networks. BMC Bioinform 8: 311. 10.1186/1471-2105-8-311 PMC2001200 17723146

[61] Guimera R, Sales-Pardo M, Amaral LAN (2004) Modularity from fluctuations in random graphs and complex networks. Physical Review E 70: 025101. 10.1103/PhysRevE.70.025101 PMC2441765 15447530

[62] Newman MEJ, Girvan M (2004) Finding and evaluating community structure in networks. Physical Review E 69: 026113. 10.1103/PhysRevE.69.026113 14995526

[63] Rousseau-Ralliard D, Goirand F, Tardivel S, Lucas A, Algaron F, et al (2010) Inhibitory effect of αS1- and αS2-casein hydrolysates on angiotensin I-converting enzyme in human endothelial cells in vitro, rat aortic tissue ex vivo, and renovascular hypertensive rats in vivo. J Dairy Sci 93: 2906–2921. 20630208 10.3168/jds.2010-3060

[64] Harary L, Farley B (1960) In vitro studies of single isolated beating heart cells. Science 131: 1674–1675. 14399673 10.1126/science.131.3414.1674

[65] Sreejit P, Kumar S, Verma R (2008) An improved protocol for primary culture of cardiomyocyte from neonatal mice. In Vitro Cell Dev Biol: Anim 44: 45–50. 18297366 10.1007/s11626-007-9079-4

[66] Rohr S, Scholy D (1991) Patterned growth of neonatal rat heart cells in culture:morphological and electrophysiological characterization. Circ Res 68: 114–130. 1984856 10.1161/01.res.68.1.114

