# Supplementary figures and images for: Therapeutic Effects of Astragaloside IV on Myocardial Injuries: Multi-Target Identification and Network Analysis
Source: PLoS One. 2012 Sep 17;7(9):e44938. doi: 10.1371/journal.pone.0044938 (PMC3444501; doi:10.1371/journal.pone.0044938)

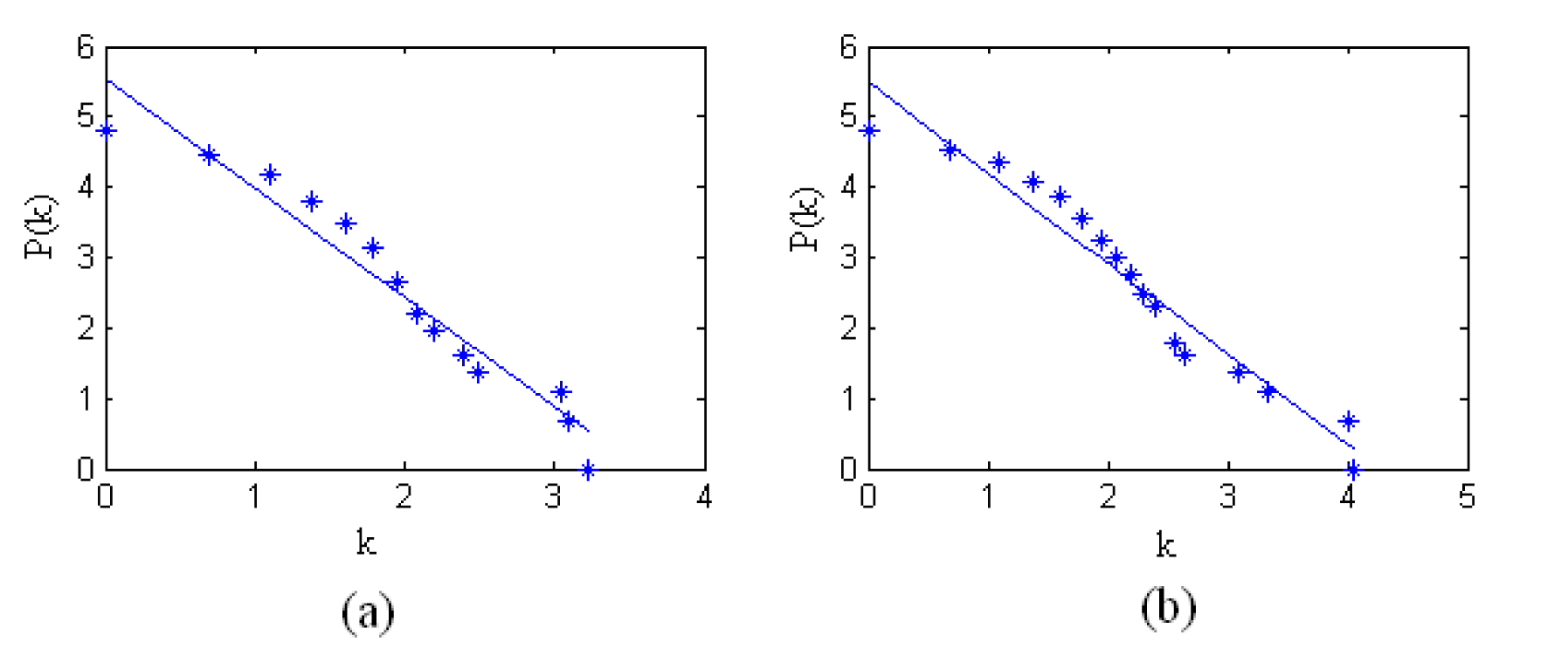

Supplement: Figure S1 — Log-log plots for degree distribution of pathway nodes in target-pathway network (a) and drug-pathway network (b) for FDA-approved small molecule CVD drugs. Distribution of pathway nodes in both of the networks obeyed power laws. (TIF) [file pone.0044938.s001.tif]

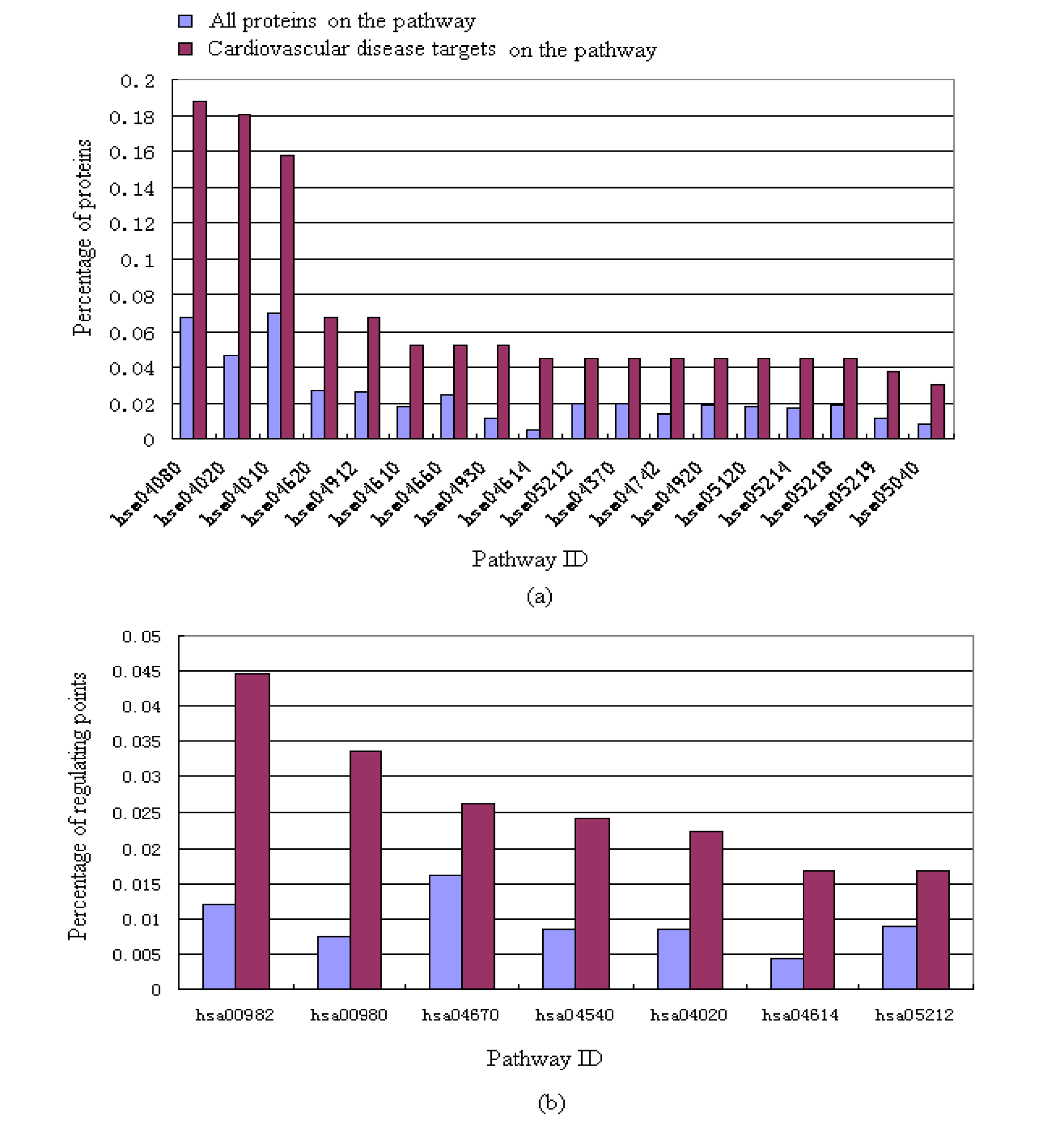

Supplement: Figure S2 — Comparison of percentage of distinct protein targets and regulating points for cardiovascular targets significantly enriched pathways (P<0.05). Pathways permuted decreasingly according to percentage. (a) Type I enrichment pathways: enrichment of distinct CVD drug targets (b) Type II enrichment pathways: enrichment of CVD drug target nodes. Pathway names are listed in Table S1. (TIF) [file pone.0044938.s002.tif]

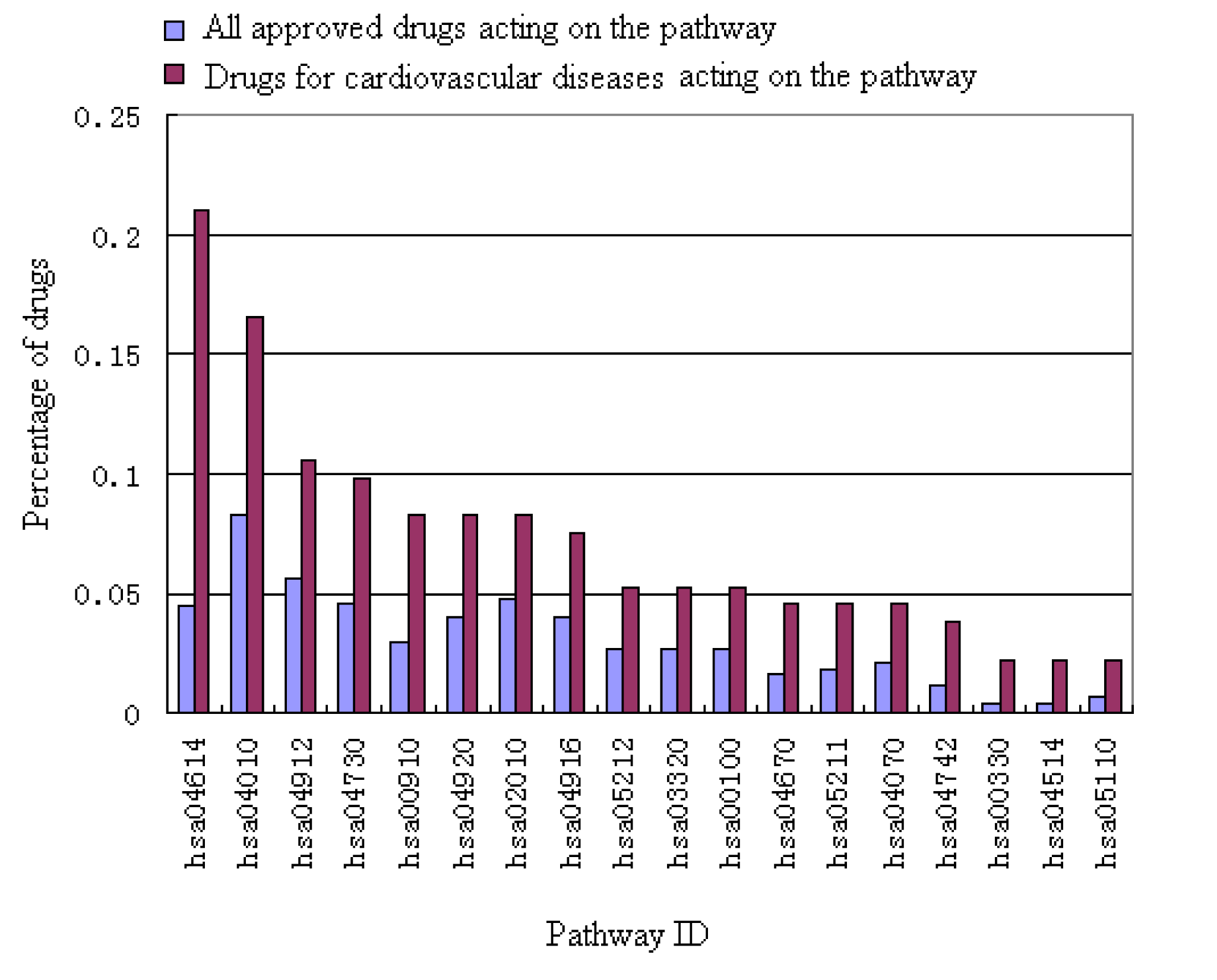

Supplement: Figure S3 — Comparison of percentage of FDA-approved drugs acting on cardiovascular drugs significantly enriched pathways (P<0.05). Pathways permuted decreasingly according to percentage. Pathway names are listed in Table S1. (TIF) [file pone.0044938.s003.tif]

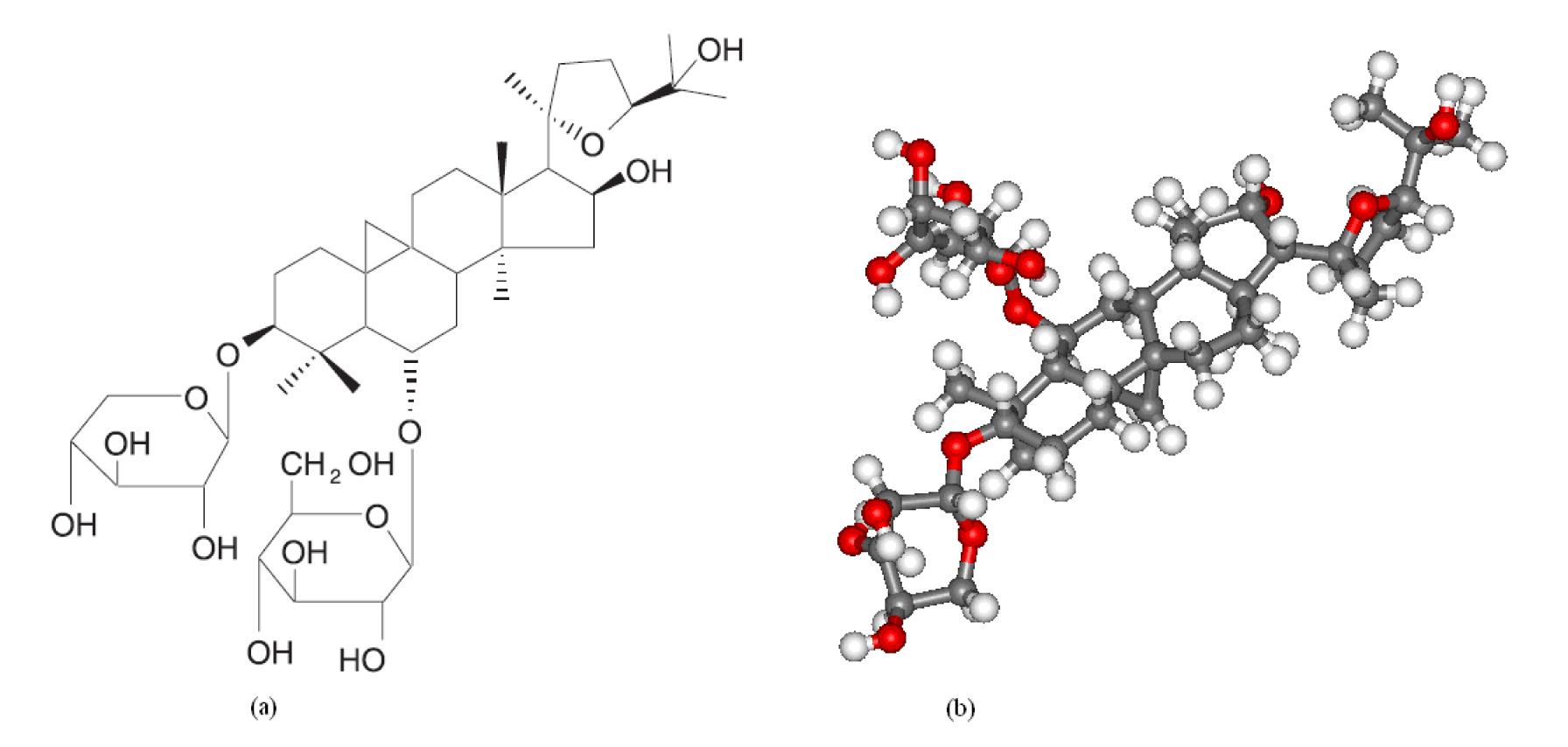

Supplement: Figure S4 — Chemical structure and 3D molecular structure of Astragaloside IV. (a) Chemical structure of AGS-IV. (b) 3D molecular structure of AGS-IV. Grey, red, and white colors represent carbon, oxygen and hydrogen atom, respectively. (TIF) [file pone.0044938.s004.tif]
