# Supplementary material for: Therapeutic Effects of Astragaloside IV on Myocardial Injuries: Multi-Target Identification and Network Analysis
Source: PLoS One. 2012 Sep 17;7(9):e44938. doi: 10.1371/journal.pone.0044938 (PMC3444501; doi:10.1371/journal.pone.0044938)
Supplement: Table S1 — List of 33 KEGG pathways that are significantly regulated by CVD drugs. (PDF) [file pone.0044938.s005.pdf]

Pathway Class Level I	Pathway Class Level II	Pathway Name	Pathway ID	Enrichment Type
Cellular Processes	Cell Communication	Gap junction	hsa04540	II
	Endocrine System	PPAR signaling pathway	hsa03320	III
		Adipocytokine signaling pathway	hsa04920	I,III
		GnRH signaling pathway	hsa04912	I,III
		Melanogenesis	hsa04916	III
		Renin-angiotensin system	hsa04614	I,II,III
	Immune System	Complement and coagulation cascades	hsa04610	I
		Leukocyte transendothelial migration	hsa04670	II,III
		T cell receptor signaling pathway	hsa04660	I
		Toll-like receptor signaling pathway	hsa04620	I
	Nervous System	Long-term depression	hsa04730	III
	Sensory System	Taste transduction	hsa04742	I,III
Environmental Information Processing	Membrane Transport	ABC transporters - General	hsa02010	III
	Signal Transduction	Calcium signaling pathway	hsa04020	I,II
		MAPK signaling pathway	hsa04010	I,III
		Phosphatidylinositol signaling system	hsa04070	III
		VEGF signaling pathway	hsa04370	I
	Signaling Molecules and Interaction	Cell adhesion molecules (CAMs)	hsa04514	III
		Neuroactive ligand-receptor interaction	hsa04080	I
Human Diseases	Cancers	Bladder cancer	hsa05219	I
		Glioma	hsa05214	I
		Melanoma	hsa05218	I
		Pancreatic cancer	hsa05212	I,II,III
		Renal cell carcinoma	hsa05211	III
	Infectious Diseases	Cholera - Infection	hsa05110	III
		Epithelial cell signaling in Helicobacter pylori infection	hsa05120	I
	Metabolic Disorders	Type II diabetes mellitus	hsa04930	I
	Neurodegenerative Diseases	Huntington's disease	hsa05040	I
Metabolism	Amino Acid Metabolism	Arginine and proline metabolism	hsa00330	III

	Energy Metabolism	Nitrogen metabolism	hsa00910	III
	Lipid Metabolism	Biosynthesis of steroids	hsa00100	III
	Xenobiotics Biodegradation and Metabolism	Drug metabolism - cytochrome P450	hsa00982	II
		Metabolism of xenobiotics by cytochrome P450	hsa00980	II
