# Supplementary material for: Therapeutic Effects of Astragaloside IV on Myocardial Injuries: Multi-Target Identification and Network Analysis
Source: PLoS One. 2012 Sep 17;7(9):e44938. doi: 10.1371/journal.pone.0044938 (PMC3444501; doi:10.1371/journal.pone.0044938)
Supplement: Table S2 — Putative therapeutic targets of AGS-IV identified from INVDOCK search of candidate protein 3D structure data set. PDB ID is the identification number for a protein in the PDB. Target Class: AC: target for approved CVD drugs; A: target for approved drugs of other classes; E: target for experimental drugs; U: protein not applied as target. The properties of proteins with no references were obtained from the Uniprot database. Proteins that have been reported in the literature as associated with CVD therapy are indicated in bold. (PDF) [file pone.0044938.s006.pdf]

Item	Gene ID	Protein Definition	PDB ID	Target Class	Gene Ontology (GO)	Properties and implications for the therapy of cardiovascular diseases
1	**ACE**	Angiotensin-converting enzyme	1o86<br>1uze<br>2c6f<br>2iul	AC	Regulation of vasoconstriction<br>Regulation of vasodilation	ACE inhibitors are widely used in the treatment of cardiovascular diseases, including congestive heart failure, coronary artery disease and hypertension[1].
2	**CALM1**	Calmodulin	1lvc	AC	Response to calcium ion	Calmodulin mediates the control of a large number of enzymes and other proteins by calcium ions which are key intracellular messengers in the cardiovascular system[1].
3	CYP3A4	Cytochrome P450 3A4	2v0m	AC	Oxidation reduction	CYP3A4 is a member of cytochrome P450 family, which is involved in the biotransformation of a diverse range of xenobiotics, including therapeutic drugs and toxins. CYP3A4 in the liver metabolizes over 50% of drugs [2].
4	**F2**	Prothrombin	1awh<br>2b5t	AC	Positive regulation of blood coagulation	Drug target of anticoagulants. Involved in blood clotting cascade [1].
5	**NOS3**	Nitric oxide synthase, endothelial	1m9k<br>3nos	AC	Positive regulation of vasodilation<br>Regulation of systemic arterial blood pressure by endothelin	*In vivo* models of myocardial infarction suggest that NOS3 overexpression can limit compensatory hypertrophy in the remote myocardium and preserve left ventricular performance. The development of therapeutic strategies designed to enhance NO signaling in cardiac myocytes may target maladaptive left ventricular remodeling and improve functional recovery after myocardial infarction[3].
6	**SERPINC1**	Antithrombin-III	2b5t	AC	Blood coagulation	Drug target of anticoagulants. Involved in blood coagulation cascade [1].
7	**EGFR**	Epidermal growth factor receptor	1ivo	AC	Positive regulation of MAP kinase activity	Activation of the EGFR causes activation of PI3K and JNK pathways, which play fundamental roles in the regulation of myocardial contractility and hypertrophy. Blockade of the EGFR attenuates Ang II-mediated cardiac hypertrophy[4].
			1mox			
8	**EGF**	Pro-epidermal growth factor	1ivo	A	Positive regulation of EGFR activity	EGF positively regulates epidermal growth factor receptor (EGFR) activity and activates MAPK activity. Inhibition of EGFR or MAPK has beneficial effects for cardiovascular

					Activation of MAPK activity	diseases[4,5].
9	ADH5	Alcohol dehydrogenase class-3	1teh	A	Oxidation reduction<br>Response to redox state	ADH5 catalyzes the oxidation of long-chain primary alcohols and the oxidation of S-(hydroxymethyl) glutathione.
10	AKR1C4	Aldo-keto reductase family 1 member C4	2fvl	A	Oxidation reduction	Catalyzes the transformation of the potent androgen dihydrotestosterone (DHT) into the less active form.
11	**CN**	Calcineurin	1m63<br>1mf8	A	Calcium ion binding<br>Calmodulin binding	Inhibition of calcineurin has proved to be effective for the treatment of cardiac hypertrophy[6].
12	**FGFR1**	Basic fibroblast growth factor receptor 1	1agw	A	MAPKKK cascade	Fibroblast growth factor 2 (FGF2) plays an important role in inducing cardiac hypertrophy. Many of the effects of FGF-2 are mediated by binding and activating FGFR. The FGF2-FGFR1 axis needs to be considered as a potential target for the management of hypertrophy[7].
13	GAPDH	Glyceraldehyde-3-phosphate dehydrogenase	1u8f	A	Oxidation reduction	Independent of its glycolytic activity, it is also involved in membrane trafficking in the early secretory pathway.
14	**HLA-DRB1**	HLA class II histocompatibility antigen, DRB1-1 beta chain	1bx2	A	Immune response	Human leukocyte antigens (HLA) are the designation for antigens in the major histocompatibility complex (MHC), a major determinant of the immune response. It is considered to play a significant role in the pathophysiology of atherosclerosis[8].
15	**INSR**	Insulin receptor	1ir3<br>2hr7	A	Activation of MAPK activity	INSR binds insulin and mediates metabolic functions of insulin. Can activate PI3K either directly by binding to the p85 regulatory subunit, or indirectly via IRS1. PI3K signaling plays a fundamental role in the regulation of myocardial contractility and hypertrophy[9].
16	KIT	Mast/stem cell growth factor receptor	1t46	A	Transmembrane receptor protein tyrosine kinase signaling pathway	This is the receptor for stem cell factor (mast cell growth factor). It has a tyrosine-protein kinase activity. Binding of ligands leads to the autophosphorylation of KIT and its association with substrates such as phosphatidylinositol 3-kinase (PI3K).

17	LCK	Proto-oncogene tyrosine-protein kinase LCK	1x27	A	Release of sequestered calcium ion into cytosol	Tyrosine kinase that plays an essential role in the selection and maturation of developing T-cell in the thymus and mature T-cell function.
18	**MME**	Neprilysin	1r1i	A	Cell-cell signaling	MME is implicated in the pathogenesis of arterial hypertension, congestive heart failure, left ventricular remodeling after myocardial infarction and other cardiovascular diseases. It is a novel target for the treatment of heart diseases[10].
19	**NQO1**	NAD(P)H dehydrogenase [quinone] 1	1d4a<br>1dxo	A	Nitric oxide biosynthetic process<br>Oxidation reduction	NQO1 is critically involved in the detoxification of xenobiotics as well as ROS and might function as an effective O^{+}_{2} scavenger in cardiac cells. Oxidative stress plays critical roles in the development of various forms of cardiovascular disorders, including myocardial ischemia-reperfusion injury, congestive heart failure, coronary arterial atherosclerosis, and chemical induced cardiotoxicity. Activation of NOQ1 increases resistance to oxidative cardiac cell injury[11].
20	OAT	Ornithine aminotransferase, mitochondrial	1gbn<br>1oat<br>2can<br>2oat	A	Ornithine-oxo-acid transaminase activity	Enzyme associated with amino-acid biosynthesis.
21	RXRB	Retinoic acid receptor RXR-beta	1h9u	A	Regulation of transcription, DNA-dependent	Nuclear hormone receptor. Involved in the retinoic acid response pathway.
22	**BST1**	ADP-ribosyl cyclase 2	1isg	E	Humoral immune response	ADPR-cyclase synthesizes cyclic ADP-ribose, a second messenger that elicits calcium release from intracellular stores. BST1 is an important mediator of cardiac hypertrophy, and inhibition of ADPR-cyclase attenuates angiotensin II-induced cardiac hypertrophy [12].

23	DAPK1	Death-associated protein kinase 1	1ig1<br>1jks	E	Induction of apoptosis by extracellular signals<br><br>Calmodulin binding	DAPK is a calmodulin-regulated serine/threonine protein kinase implicated in diverse apoptosis pathways, including those involved in neuronal cell death and tumour suppression. DAPK could be a potential therapeutic target for diseases characterized by rapid neurodegeneration, such as stroke or traumatic brain injury[13].
24	GCK	Glucokinase	1v4s	E	Positive regulation of insulin secretion	GCK intervenes in the regulation of glucose metabolism[14].
25	**PIK3CG**	Phosphoinositide 3-kinase gamma	1e8z<br>2a4z	E	G-protein coupled receptor protein signaling pathway	PIK3CG appears to negatively control cardiac contractility, thus becoming a possible drug target for the treatment of critical human cardiac pathologies, such as infarction or heart failure[15].
26	**MAPK12 (ERK6)**	Mitogen-activated protein kinase p38 gamma	1cm8	E	MAP kinase activity<br><br>Ras protein signal transduction	It is highly likely that p38 and JNK are both required to generate a hypertrophic or apoptotic response in overloaded hearts. It has been known that MAP kinases are sensitive to oxidative stress and antioxidants preferentially inhibit JNK and p38 [5].
27	**MAPK14**	Mitogen-activated protein kinase p38 alpha	1wbv	E	MAP kinase activity<br>Ras protein signal transduction	See MAPK12 (Item 26).
28	**MAPK8 (JNK1)**	c-Jun N-terminal kinase 1	2no3	U	JUN kinase activity<br>JUN phosphorylation	See MAPK12 (Item 26).
29	**ARF1**	ADP-ribosylation factor 1	1hur	U	GTPase activity<br>Small GTPase mediated signal transduction	Arfs are Ras-related small GTP-binding proteins[16] that are implicated in regulation of endothelial function, smooth muscle cell contraction, proliferation, and migration, as well as cardiomyocyte hypertrophy. Targeting small G proteins could constitute promising therapeutic approaches in cardiovascular disorders[17].
30	DAPK2	death-associated protein kinase 2	2a2a	U	Induction of apoptosis by extracellular signals<br>Calmodulin binding	See DAPK1 (Item 23).

					Calmodulin-dependent protein kinase activity	
31	**mTOR**	Mammalian target of rapamycin	1nsg<br>2fap<br>4fap	U	Phosphoinositide 3-kinase complex<br>Protein serine/threonine kinase activity	mTOR functions in the regulation of cardiac growth. Inhibition of mTOR attenuates the development of pressure overload cardiac hypertrophy and regresses established cardiac hypertrophy[18].
32	**HLA-A**	HLA class I histocompatibility antigen, a-11 alpha chain	1x7q	U	Immune response	See HLA-DRB1 (Item 14).
33	**HLA-DRA**	HLA class II histocompatibility antigen, dr alpha chain	1bx2<br>1seb<br>2icw	U	Immune response	See HLA-DRB1 (Item 14).
34	**IRAK4**	Interleukin-1 receptor-associated kinase 4	2oib	U	I-kappaB kinase/NF-kappaB cascade	IRAK-4 plays a central role in mediating NFκB activation and innate immunity signaling and thus could be potential target for inflammatory diseases[19].
35	**MMP1**	Interstitial collagenase	4ayk	U	Metalloendopeptidase activity calcium ion binding	MMP activity contributes to left ventricular (LV) dilation and progression to LV dysfunction and direct MMP inhibition can attenuate this process [20].
36	MSN	Moesin	1ef1	U	Leukocyte adhesion<br><br>Leukocyte migration	Probably involved in connections of major cytoskeletal structures to the plasma membrane.
37	**NCF1**	Neutrophil NADPH oxidase factor 1	1o7k	U	Innate immune response<br><br>superoxide release	NADPH oxidase has emerged as a major source of oxidative stress in the artery wall, particularly in artery disease. Nitric oxide and targeted inhibitors of NADPH oxidase are more likely to prevent the deterioration of vascular function that leads to stroke and heart attack, than are conventional antioxidants[21].

38	**RAC3**	RAS-related C3 botulinum toxin substrate 3	2g0n<br>2ic5	U	GTPase activity<br>Small GTPase mediated signal transduction	Rac3 is a member of the Rho family of small GTP-binding proteins. Small G proteins are implicated in regulation of endothelial function, smooth muscle cell contraction, proliferation and migration, as well as cardiomyocyte hypertrophy. Targeting small G proteins could constitute promising therapeutic approaches in cardiovascular disorders[17].
39	**TGFA**	Protransforming growth factor alpha	1mox	U	Activation of MAPK activity<br>Positive regulation of EGFR activity	TGFA is able to bind to the EGF receptor to regulate epidermal growth factor receptor (EGFR) activity. It is also able to activate MAPK activity. Inhibition of EGFR or MAPK has beneficial effects for cardiovascular diseases[4,5].

Reference

1. Maglott D, Ostell J, Pruitt KD, Tatusova T (2005) Entrez Gene: gene-centered information at NCBI. Nucleic Acids Res: D54 - 58.
2. Guengerich F (1999) Cytochrome P450 3A4: regulation and role in drug metabolism. Annu Rev Pharmacol Toxicol 39: 1-17.
3. Bloch KD, Janssens S (2005) Cardiomyocyte-Specific Overexpression of Nitric Oxide Synthase 3: Impact on Left Ventricular Function and Myocardial Infarction. Trends in Cardiovascular Medicine 15: 249-253.
4. Shah B, KJ C (2003) A central role of EGF receptor transactivation in angiotensin II -induced cardiac hypertrophy. Trends in Pharmacological Sciences 24: 239-244.
5. Pandya N, Santani D, Jain S (2005) Role of mitogen-activated protein (MAP) kinases in cardiovascular diseases. Cardiovasc Drug Rev 23: 247-254.
6. Sheng H, Zhu J, Wu X, Zhang J (2008) Blockade of calcineurin reverses cardiac hypertrophy and induces the down-regulation of JNK mRNA expression in renovascular hypertensive rats. Journal of Renin-Angiotensin-Aldosterone System 9: 139 - 145.
7. Kardami E, Jiang Z-S, Jimenez SK, Hirst CJ, Sheikh F, et al. (2004) Fibroblast growth factor 2 isoforms and cardiac hypertrophy. Cardiovasc Res 63: 458-466.
8. Diamantopoulos EJ, Andreadis EA, Vassilopoulos CV, Vlachonikolis IG, Tarassi KE, et al. (2003) HLA phenotypes as promoters of cardiovascular remodelling in subjects with arterial hypertension. J Hum Hypertens 17: 63-68.
9. Oudit GY, Sun H, Kerfant B-G, Crackower MA, Penninger JM, et al. (2004) The role of phosphoinositide-3 kinase and PTEN in cardiovascular physiology and disease. Journal of Molecular and Cellular Cardiology 37: 449-471.
10. De Vos J, Thykjaer T, Tarte K, Ensslen M, Raynaud P, et al. (2002) Comparison of gene expression profiling between malignant and normal plasma cells with oligonucleotide arrays. Oncogene 21: 6848-6857.
11. Cao Z, Zhu H, Zhang L, Zhao X, Zweier JL, et al. (2006) Antioxidants and Phase 2 Enzymes in Cardiomyocytes: Chemical Inducibility and Chemoprotection Against Oxidant and Simulated Ischemia-Reperfusion Injury. Experimental Biology and Medicine 231: 1353-1364.
12. Gul R, Park J-H, Kim S-Y, Jang KY, Chae J-K, et al. (2009) Inhibition of ADP-ribosyl cyclase attenuates angiotensin II-induced cardiac hypertrophy. Cardiovasc Res 81: 582-591.
13. Schumacher A, Velentza A, Watterson D (2002) Death-associated protein kinase as a potential therapeutic target. Expert Opin Ther Targets 6: 497-506.
14. Mordier S, Iynedjian PB (2007) Activation of mammalian target of rapamycin complex 1 and insulin resistance induced by palmitate in hepatocytes. Biochemical and Biophysical Research Communications 362: 206-211.
15. Alloatti G, Montrucchio G, Lembo G, Hirsch E (2004) Phosphoinositide 3-kinase gamma: kinase-dependent and -independent activities in cardiovascular function and disease. Biochem Soc Trans 32: 383-386.
16. Alfarano C, Andrade CE, Anthony K, Bahroos N, Bajec M, et al. (2005) The Biomolecular Interaction Network Database and related tools 2005 update. Nucleic Acids Res 33: D418-424.
17. Barandier C, Ming XF, Yang ZH (2003) Small G proteins as novel therapeutic targets in

cardiovascular medicine. News Physiol Sci 18: 18-22.
18. McMullen J, Sherwood M, Tarnavski O, Zhang L, Dorfman A, et al. (2004) Inhibition of mTOR signaling with rapamycin regresses established cardiac hypertrophy induced by pressure overload. Circulation 109: 3050-3055.
19. Ringwood L, Li L (2008) The involvement of the interleukin-1 receptor-associated kinases (IRAKs) in cellular signaling networks controlling inflammation. Cytokine 42: 1-7.
20. Peterson JT, Hallak H, Johnson L, Li H, O'Brien PM, et al. (2001) Matrix Metalloproteinase Inhibition Attenuates Left Ventricular Remodeling and Dysfunction in a Rat Model of Progressive Heart Failure. Circulation 103: 2303-2309.
21. Dusting GJ, Selemidis S, Jiang F (2005) Mechanisms for suppressing NADPH oxidase in the vascular wall. Mem Inst Oswaldo Cruz, Rio de Janeiro 100(Suppl. I): 97-103.
